# A qualitative study exploring the barriers to attending structured education programmes among adults with type 2 diabetes

**DOI:** 10.1186/s12913-022-07980-w

**Published:** 2022-04-30

**Authors:** Imogen Coningsby, Ben Ainsworth, Charlotte Dack

**Affiliations:** 1 Department of Psychology, University of Bath, Bath, BA2 7AY UK; 2 Present Address: Public Health and Preventative Services, Bath and North East Somerset Council, Keynsham Civic Centre, Market Walk, Keynsham, BS31 1FS UK

**Keywords:** Type 2 diabetes, Diabetes mellitus, Adult, Patient non-attendance, Self-management

## Abstract

**Background:**

Diabetes self-management education, a universally recommended component of diabetes care, aims to support self-management in people with type 2 diabetes. However, attendance is low (approx. 10%). Previous research investigating the reasons for low attendance have not yet linked findings to theory, making it difficult to translate findings into practice. This study explores why some adults with type 2 diabetes do not attend diabetes self-management education and considers how services can be adapted accordingly, using Andersen’s Behavioural Model of Health Service Utilisation as a framework.

**Methods:**

A cross-sectional semi-structured qualitative interview study was carried out. Semi-structured interviews were conducted by telephone with 14 adults with type 2 diabetes who had verbally declined their invitation to attend diabetes self-management education in Bath and North East Somerset, UK, within the last 2 years. Data were analysed using inductive thematic analysis before mapping the themes onto the factors of Andersen’s Behavioural Model.

**Results:**

Two main themes were identified: *‘perceived need’* and *‘practical barriers’*. The former theme explored participants’ tendency to decline diabetes education when they perceived they did not need the programme. This perception tended to arise from participants’ high self-efficacy to manage their type 2 diabetes, the low priority they attributed to their condition and limited knowledge about the programme. The latter theme, ‘*practical barriers’*, explored the notion that some participants wanted to attend but were unable to due to other commitments and/or transportation issues in getting to the venue.

**Conclusions:**

All sub-themes resonated with one or more factors of Andersen’s Behavioural Model indicating that the model may help to elucidate attendance barriers and ways to improve services. To fully understand low attendance to diabetes education, the complex and individualised reasons for non-attendance must be recognised and a person-centred approach should be taken to understand people’s experience, needs and capabilities.

**Supplementary Information:**

The online version contains supplementary material available at 10.1186/s12913-022-07980-w.

## Introduction

Type 2 diabetes has the fastest rising prevalence of any long-term condition [1]. Self-management, defined as a set of skilled behaviours one engages in to manage an illness [2], plays a central role in keeping blood glucose within a safe threshold in people with type 2 diabetes [3]. The knowledge and skills needed to self-manage one’s type 2 diabetes are diverse and include numerous daily activities, such as carbohydrate counting, exercise and self-monitoring of blood glucose levels [4]. When blood glucose levels rise above the normal threshold, this can lead to serious complications such as blindness, renal failure and amputation [5].

In the UK, diabetes self-management education (DSME) is a recommended component of diabetes care which aims to improve individuals’ knowledge, skills and confidence enabling them to self-manage their type 2 diabetes and improve self-care and clinical outcomes including glycaemic control (level of glucose in the blood) [6]. DSME has an evidence-based and theory-driven curriculum that is delivered in groups by trained educators [6]. In the UK, examples of DSME include X-PERT diabetes [7], a 6-week programme in sessions of 2.5 hours and DESMOND [8], a 1 or 2 day programme lasting 6 hours in total.

There is strong evidence that DSME confers significant benefits on self-management behaviours as well as clinical, lifestyle and psychosocial outcomes in adults with type 2 diabetes. For example, a randomised controlled trial [9] found that participation in the DSME programme, X-PERT, led to improvements in a range of outcomes at 14 month follow-up such as glycaemic control, body mass index, waist circumference, cholesterol, diabetes knowledge and psychosocial adjustment. These findings have also been replicated in a national audit of X-PERT diabetes programmes [10]. Systematic reviews with meta-analysis have also conferred similar positive effects [11, 12]. For example, a systematic review [13] of DSME for people with type 2 diabetes found that, at 1-year follow-up, patient satisfaction and body weight had significantly improved and, at 2-year follow-up, there were significant improvements in blood glucose levels and diabetes knowledge. These findings indicate that DSME is a well-supported intervention to improve clinical, lifestyle and psychosocial outcomes in adults with type 2 diabetes.

However, attendance to DSME is low [10, 14]. Approximately 90% of those invited to DSME do not attend [10]. Low DSME attendance is a major concern given that DSME can have positive effects on health outcomes [12, 13]. Moreover, individuals who do not attend or do not complete DSME are more likely to have reduced adherence to self-management activities and glycaemic control and are at a fourfold increased risk of having complications than those who attend and complete DSME [15, 16]. It is therefore paramount that the reasons for non-attendance are well-understood among health professionals, commissioners and policy makers. This will help identify ways attendance can be optimised, as well as establish the effectiveness of DSME whilst taking into consideration who does and doesn’t engage with DSME and why. A more nuanced understanding of the barriers to DSME may also help elucidate whether for some people alternative support (e.g. social/emotional/psychological support) would be more appropriate and beneficial.

Recent research has highlighted various psychosocial, cultural and practical barriers to attendance [14, 17]. For example, a recent systematic review [14] found reasons for non-attendance included logistical, medical, financial, emotional and cultural barriers, as well as a lack of perceived benefit and feeling that oneself has sufficient knowledge of their condition. Similarly, a recent qualitative report [17] reported additional barriers including a lack of knowledge of the existence and benefits of DSME among people with type 2 diabetes and their healthcare providers, and poor integration within existing healthcare.

However, the lack of application of previous findings to theory makes it difficult to interpret and translate findings into practice as there lacks a framework for identifying key areas for improvement [18]. Andersen’s Behavioural Model of Health Service Utilisation [19, 20] (ABM) may be useful to explain and predict non-attendance because it acknowledges both individual and contextual characteristics that may facilitate or impede health service utilisation. The ABM proposes that an individual’s use of health services depends on three characteristics: predisposing factors (sociocultural and psychological characteristics of individuals that exist prior to their illness such as sex, age, culture, values and attitudes towards health and health services), enabling factors (practical aspects of obtaining care such as the means and know how to access health services, income, travel, extent and quality of social relationships, knowledge about services, as well as available health personnel and waiting times) and need factors (conditions perceived by the patient or evaluated by health professionals as requiring the use of health services) [19]. ABM has been used to explain and predict the use of a range of health services required for tertiary disease management (e.g. cardiac rehabilitation [21] and HIV medication adherence [22]). Therefore, ABM is an appropriate model to help elucidate barriers to DSME attendance and identify priority areas to increase uptake.

### Study aim

This study explored reasons for non-attendance to DSME among adults with type 2 diabetes in Bath and North East Somerset, UK, as well as assess the utility of ABM as a framework for elucidating barriers to DSME attendance and highlighting key areas to improve local services.

## Methods

### Design

A semi-structured qualitative interview study was conducted. Due to the Coronavirus (COVID-19) pandemic present during data collection, all interviews were conducted via telephone. The study was conducted in accordance with the British Psychological Society recommendations for research. Ethical approval was received from the University of Bath Psychology Ethics Committee (reference number: 20–133). The COnsolidated criteria for REporting Qualitative research (COREQ) [23] was used to guide reporting (appendix A).

### Participants

Convenience sampling was used to ensure participants met the following eligibility criteria:

- Over 18 years old
- Live in Bath and North East Somerset, UK
- Diagnosed with type 2 diabetes
- Verbally declined their invite to attend the DSME programme, X-PERT, within the last 2 years

**Fig. 1:**
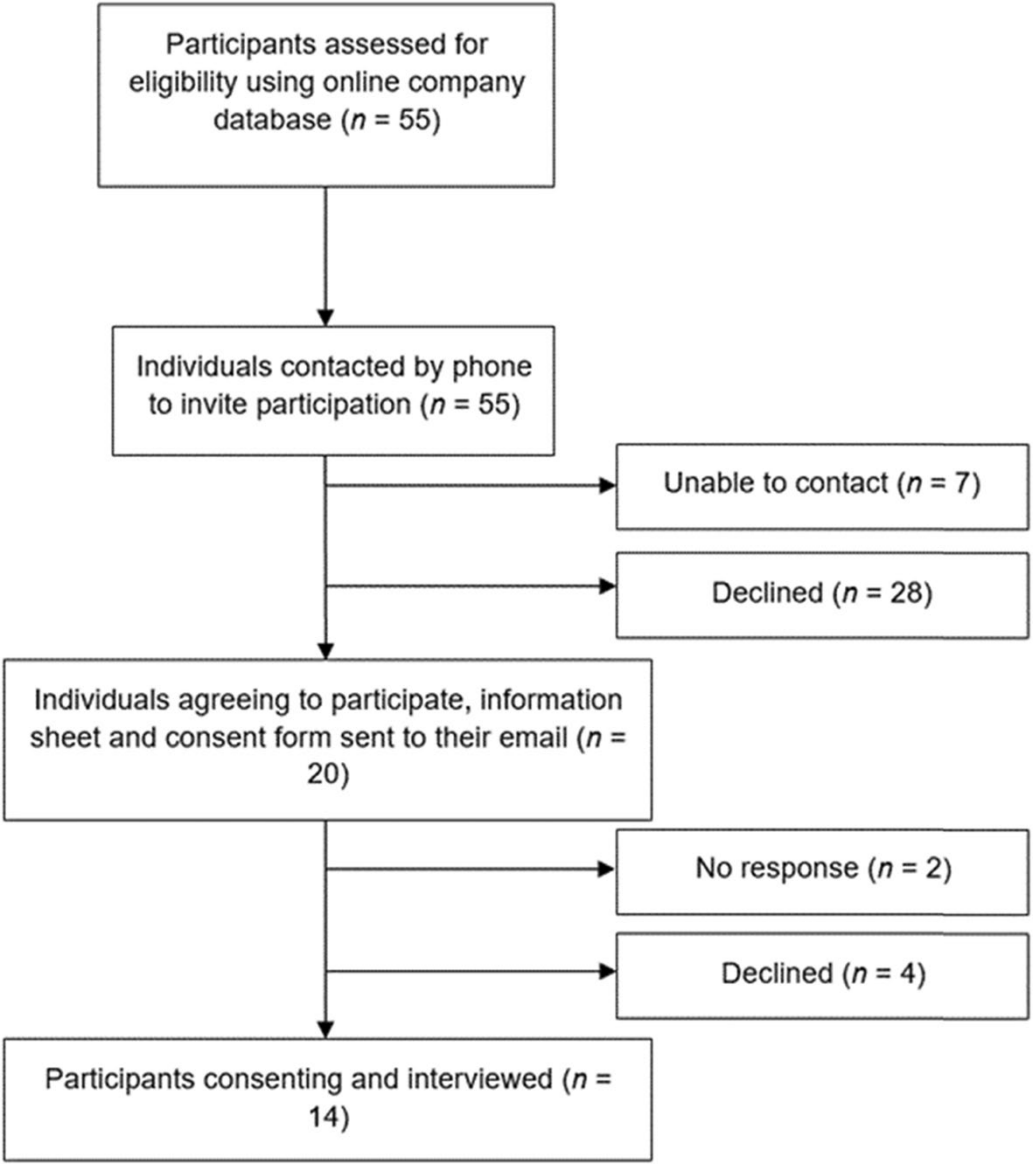
Recruitment strategy

Eligible individuals were phoned by the researcher and asked if they would be interested in participating. Out of the 55 individuals eligible to participate, 20 were willing to take part. 28 declined due to disinterest, lack of time, denial of their type 2 diabetes and beliefs that their type 2 diabetes was not important. Seven individuals could not be contacted. Prospective participants were sent an information sheet and consent form via email. Two participants did not respond, and four participants declined due to lack of time. In total, 14 participants gave written and verbal informed consent and were interviewed (Fig. 1).

Of the 14 participants, five were women and nine were men. Participants were aged between 27 and 80 years (*M* = 57, *SD* = 15.5). The length of time since diagnosis varied between 3 months and 40 years (*M* = 4.6 years). According to participants’ BMI, three participants were classified as overweight, nine were obese and one was extremely obese (see Table 1 for participants’ demographics).

**Table 1:** Participant Demographics

Pseudonym	Age	Gender	Time since diagnosis	Body Mass Index
Alice	30	Female	7 months	42.4 (extremely obese)
Julie	60	Female	3 months	31.0 (obese)
Harry	64	Male	2 years	26.2 (overweight)
John	74	Male	8 months	30.1 (obese)
Shelley	56	Female	10 months	34.7 (obese)
Andrew	60	Male	2–3 years	30.0 (obese)
Mark	50	Male	8 months	26.3 (overweight)
Derek	79	Male	4–5 years	31.9 (obese)
Edward	80	Male	40 years	Unwilling to provide
Alex	50	Male	6 years	30.5 (obese)
Graham	54	Male	1 year	30.1 (obese)
Becky	27	Female	1.5 years	36.7 (obese)
Lisa	57	Female	4 years 8 months	26 (overweight)
Robert	57	Male	1.5 years	31.3 (obese)

### Procedure

Interviews were conducted by telephone in June 2020 by author IC, a female Health Psychology Masters student who had received formal training in qualitative research. The interviewer had no previous relationship with participants and started each interview by thanking the participant for their time and providing an overview of the research and its aims. Participants were given the opportunity to ask questions before being asked to give verbal informed consent. After obtaining informed consent the interview commenced with only the interviewer and participant present. All individuals who participated in this study gave informed consent prior to participation.

A semi-structured interview schedule (developed by author IC) was used to flexibly guide the interviews. It was piloted and adapted during the first three interviews to ensure questions were clear and not misinterpreted. The question “what would your ideal diabetes programme look like?” was discarded as participants reported being unable to answer due to their limited understanding of DSME. The final interview schedule had two main topics reflecting the aims of this study: 1) Barriers to attending DSME; and 2) Programme improvements (appendix B). Interviews lasted, on average, 31 minutes (shortest = 15; longest = 52), and were audio-recorded.

After the interview, participants were thanked for their time, verbally debriefed and given the opportunity to ask any further questions before being emailed a debrief sheet. The interview was then transcribed verbatim, anonymized and imported into the software package QSR NVivo to record field notes and facilitate data analysis. Transcripts were not returned to participants for comment or correction.

### Analytic approach

Data were analysed using reflexive thematic analysis to enable the identification of patterns/themes related to barriers to DSME attendance [24]. An inductive approach was used whereby interviews were exploratory and analysis was driven by participants’ accounts [25]. In the final interviews, no new major insights emerged from the data. Therefore, it was believed data saturation had been reached [26].

To improve rigour, Braun and Clarke’s six phases of thematic analysis [25] were used to generate themes from the data. In accordance with their recommendations, analysis was iterative, moving bi-directionally through the phases to provide a thorough analysis of the data. Analysis began with data familiarisation whereby author IC listened to the audio files and read the transcripts several times. IC then generated codes inductively in NVivo before collating the codes into provisional themes by considering their different relationships, similarities and differences. Authors BA and IC reviewed provisional themes by checking each code and its associated data fit with the theme before defining and naming the themes. Sub-themes were created where themes were large or incorporated nuances in meaning within the data. Participants were not asked for feedback on the findings.

After the final themes had been agreed, sub-themes were mapped onto the factors of ABM (Table 2). This required the researchers to re-read data within the sub-themes and assign the sub-themes to appropriate factors. Occasionally, data coded under one sub-theme could be assigned to more than one factor. All sub-themes could be applied to at least one factor. This approach ensured the analysis was data driven and meant that the robustness of ABM in explaining the data could be tested against the themes during this mapping process.

**Table 2:** Themes and sub-themes

Theme	Sub-theme	Construct of ABM
1. Perceived need	Self-efficacy to manage diabetes	Predisposing, Enabling, Need
	Low prioritisation of diabetes	Predisposing, Need
	Limited knowledge of DSME	Predisposing, Enabling, Need
2. Practical barriers	Programme schedule	Enabling
	Transportation issues	Predisposing, Enabling

## Results

Two themes, in relation to barriers to attendance, were identified; ‘*perceived need’* and *‘practical barriers’.* Each theme had several sub-themes (Table 2). Illustrative quotes are referenced using pseudonyms (Table 1).

### Theme 1: perceived need

A key reason for not attending DSME was individuals’ perception they did not need to attend DSME: *“I don’t really need this”* (John). This was evident among those who had high self-efficacy to manage their type 2 diabetes (sub-theme 1), attributed a low priority to their type 2 diabetes (sub-theme 2) and/or had limited knowledge about DSME (sub-theme 3).

#### Self-efficacy to manage diabetes

Two frequently reported reasons for declining DSME were already being well informed and having acquired sufficient knowledge about diabetes and how to manage it. This negated the need to attend DSME.

*“I wasn’t sure that it would contribute anything. I know all about controlling diet and controlling blood sugars. I mean I’ve seen it in spades, and did I want to hear it all again? Well no not really.”* (Edward)

There was a sense of animosity and monotony towards attending as individuals did not want to listen to information they had already heard and knew.

*“I wasn’t going to sit with ten other people just sitting listening to someone preaching to me what I should and shouldn't do when I already know what I should and shouldn't do.”* (Alex)

For some, there was a sense of pleasure by declining the course because it provided a space to others who “*needed it more*” (Alex). They perceived themselves as highly knowledgeable of their condition and as differing from others who may be less knowledgeable and therefore more in need of support. They explained non-attendance as an altruistic act of freeing up space for another individual.

*“I did already know it all! So to me it just seemed like a waste of everybody's time and a valuable space for somebody who really needed it.”* (Julie)

Participants reported engaging in a variety of activities to manage their type 2 diabetes such as dieting, exercising and testing their blood glucose. Improvements in biomedical indices, such as blood pressure, cholesterol and glycaemic level, led to a sense of personal achievement and gave individuals credible and objective evidence that their type 2 diabetes was under control, and thus, did not warrant the need for them to seek additional help.

*“I felt like I was making apparently decent progress. Not only the sugar levels had dropped, but I improved my cholesterol and blood pressure, so I felt like I was controlling things reasonably well”* (Mark)

In addition to biomedical indices, perceived disease severity was also influenced by physicians’ assessment of one’s type 2 diabetes. Several participants described being reassured by their GP or nurse that their type 2 diabetes was not harmful at the current time. This positive assessment and framing of their condition as *‘borderline’* (Mark) or ‘*marginal’* (Andrew), as stated by some participants, led people with type 2 diabetes to believe, and perhaps correctly so, that they did not need to take further action.

*“They [the nurses] don’t seem to be too worried about me and my condition. They can’t see any danger signals like “you got to do something really serious about this now because your body is telling us that things don’t look too good”. So really I haven’t been worried enough to think oh I ought to do something.”* (John)

One reason for not attending DSME arose from the pre-existing receipt of adequate support and care from their physician. Participants did not feel the need to seek out additional education as they were already satisfied with the care they were receiving.

*“I just thought well I’m quite happy with the way I’m being looked after thank you very much and that’s the reason I didn’t want to get too involved.”* (Harry)

In many instances, individuals used regular healthcare appointments with their physician to monitor their condition and inform them if further action is required. This suggests some individuals do not see self-management as only their responsibility, but a set of skills to be built collaboratively with a health care professional.

“*I will go and see the nurse every so often who does the annual “you’ve got worse or better”. I'm kind of in the rota now for every so often going along and being listened to and talked to as well about things that I might need to do or things that perhaps might be getting worse.”* (Julie)

#### Low prioritisation of diabetes

Another barrier to attending DSME was that their type 2 diabetes took a low priority with some participants saying it was *“just another thing”* and “*not very important*” (Derek). This was particularly common among those who had one or more comorbidities. Individuals with comorbidities considered their comorbidity as more important or requiring more attention than their type 2 diabetes.

*“I’ve got other health issues that are more demanding at the moment to me and affect me rather than type 2 diabetes which is just something else and I don’t really need it.”* (John)

The low priority some participants attributed to their type 2 diabetes may reflect attempts to avoid or deny their condition by minimising its importance. Indeed, one participant stated going through a *“period of denial”* (Lisa) in the early stages of her diagnosis. Avoidance and denial are coping strategies that protect an individual from the stress of an external experience, in this case, being diagnosed and living with type 2 diabetes [27]. Attending DSME might challenge this coping strategy if they perceive they will be exposed to the reality of their own stress.

For some individuals, the low prioritisation of their type 2 diabetes arose from their fatalistic attitudes towards their condition. Fatalistic attitudes (beliefs something is predetermined and one is powerless to change [28]) were most common in older participants. For example, Derek (age 79) stated “*my sell by date has gone for that [DSME]. I feel like it’s too late*”, illustrating that some older individuals believe that lifestyle changes made as a result of attending DSME would have limited benefit on their type 2 diabetes and that attendance is a waste of time. Similarly, another participant stated:

*“I was approaching eighty and if the X-PERT programme was trying to prolong my life I acknowledged that I didn’t have much that you could prolong”* (Edward, 80 years old)

Together these extracts illustrate that some individuals with type 2 diabetes may weigh up the time spent attending DSME against the time gained from attending to inform their decision about whether attendance would be worthwhile.

#### Limited knowledge of DSME

Another key barrier to attendance was limited information about the content of the sessions, the reason for their referral or the personal benefits of attending DSME. This prevented individuals from deciding whether they needed to attend as they were unable to identify the purpose of DSME or what they could gain.

*“I got a leaflet through the post about it [DSME] so I kind of understood what it was about in general terms I think but I wasn’t really sure what each of the sessions would address. It would be nice to know and understand a bit more about it.”* (Andrew)

Nearly all participants who reported having limited knowledge about DSME stated they would be more likely to attend if they had received more information about what they could gain from attending. This suggests that perceiving positive benefits from participating in DSME may play an important role in people’s decision to participate.

*“My GP should have given me a better understanding of what it’s going to offer me. If I was confident and convinced that it was going to help me manage things like diet and lifestyle then my mind feels like it might be beneficial”* (Andrew).

A deficit of information about DSME was typically attributed to the GP or nurse caring for the individual. This suggests that primary care physicians are a key source of information and have the potential to play an important role in an individual’s decision to attend DSME. This may also reflect individuals’ perception of a doctor-patient relationship whereby their physician is an expert whose role is to provide knowledge, inform and educate them about treatment.

*“It was like well “why are you referring me?”, “why aren’t you taking the time?”. You’re my doctor and you know my history. I think an explanation and maybe a little bit more understanding towards me as to why it was happening.”* (Becky)

*“It’s [DSME] not really something I’ve got a lot of knowledge about anyway which I think falls down to the GP. I think my GP should have explained it more”* (Alice)

### Theme 2: practical barriers

Numerous practical barriers prevented individuals from being able to attend DSME. These barriers included the *programme schedule* (sub-theme 1) and *transportation issues* (sub-theme 2).

#### Programme schedule

A frequently cited barrier to attendance were other commitments, such as work and childcare, that conflicted with the programme’s hours of operation. These commitments prevented individuals from being able to attend, despite them expressing motivation to do so.

*“I was quite interested in going, but unfortunately the times didn’t match with my work life.”* (Becky)

Some participants could not commit to attending each session. Unsurprisingly, this was common among those who worked shifts, irregular hours or abroad.

*“I had to attend the same class every week [ … ] well I don’t have that flexibility because my rota changes on a weekly basis.”* (Alice)

Several participants reported that online DSME would help them overcome barriers associated with work, childcare and commitment because it would enable them to complete the programme at their own convenience; as one participant stated: “*You don’t have to be in a particular place at a particular time.*” (Andrew).

Similarly, another barrier to attending DSME was the programme’s schedule which often impeded or interrupted individuals’ limited free time which they did not want to sacrifice. Free time was perceived highly ‘*valuable’* (Edward) and ‘*precious’* (Andrew) and attending the programme would require an investment of time which would draw away from other highly valued activities such as hobbies and family life.

*“I knew there was a weekend course and the main two things for me were taking up my time at the weekend because I work away quite a lot and I like to spend weekends with my kids.”* (Alex)

#### Transportation issues

Difficulties travelling to DSME was another impediment to attendance, particularly if the individual had work commitments or needed public transport to get to the programme’s venue.

*“A lot of it was buses and the times wouldn’t have got me back in time to go to work. Hanging about for buses … it’s just awkward.”* (Shelley)

Some individuals could not attend DSME due to a comorbidity which presented mobility and transportation issues in getting to the venue. Comorbidities cited in this study included severe back or leg problems, cancer, stroke, chronic obstructive pulmonary disease, amputation and arthritis.

*“The flare up of the rheumatoid arthritis was so bad at one point that I had difficulty pulling up my own pants so, if you can imagine, having to actually walk anywhere was very painful.”* (Julie)

For people with a physical comorbidity, attending DSME may require the aid of others to get to the venue. This was associated with logistical barriers (e.g. time and availability of carer/driver) as well as the mental burden of hassling another person. For people with mobility-limiting comorbidities, these barriers may outweigh the benefits of attending.

*“Being disabled I need to summon up Dave my driver who can take me wherever I need to go [ … ] it’s too much of a hassle [ … ] just trying to gauge how long the meeting would take and how much a question and answer would be and then getting the driver to pick me up at the right time without wasting anybody’s time is a difficult issue.”* (Edward)

## Discussion

This study conducted interviews to gather insights into the reasons for not attending DSME among adults with type 2 diabetes. One of the two main reasons for non-attendance identified in this study was participants’ perception they did not need DSME. This arose from individuals’ self-efficacy to manage their condition, the low priority they gave their type 2 diabetes and having limited information/knowledge about DSME. The second main reason for non-attendance were practical barriers and these encompassed structural issues relating to transport and the programme’s schedule. This discussion chapter will focus on how these barriers relate to the constructs in ABM and previous literature, as well as consider the ways in which attendance to DSME can be optimised.

Individuals’ self-efficacy regarding control over their type 2 diabetes and knowledge about their condition were frequently cited as reasons for non-attendance in the current study. These findings support previous research that has found perceptions of knowledge and competency in self-managing one’s type 2 diabetes as barriers to DSME attendance [29, 30]. According to ABM, ‘*predisposing factors’* which can impede health service use include high self-efficacy such as knowledge of one’s health condition and ability to manage one’s condition [19]. This construct, therefore, mirrors the findings in the current study whereby key reasons for non-attendance included having good knowledge about one’s condition and the necessary skills to self-manage their type 2 diabetes.

The present study also found that health professionals may influence individuals’ perceived need to attend DSME. Physicians who conveyed to individuals that their condition was not serious often led individuals to believe they did not need to take further action. This suggests individual factors (e.g. self-efficacy) are not the sole determinants of perceived need but external factors, such as a physician’s assessment, also play a role in individuals’ appraisal of whether they need to attend DSME. These findings are congruent with the ‘*need factors’* construct of ABM which specifically distinguishes the difference between health professionals’ and individuals’ judgement of health status and, in turn, recognises the influence health professionals can have on individuals’ engagement with a particular health service [19]. This supports the use of ABM in mapping out the reasons for not attending DSME. In light of this, it is important that health professionals accurately assess and convey to individuals the status of their health and do not undermine or over-emphasise the severity of an individual’s type 2 diabetes. Undermining the severity of an individual’s diabetes may, as our study suggests, undermine motivation to attend DSME. On the other hand, over-emphasising the severity of an individual’s type 2 diabetes may lead to undue anxiety [31]. Health professionals therefore play a key role in helping an individual understand the severity of their type 2 diabetes and, in turn, influencing individuals’ decision about whether to participate in DSME.

A novel barrier to attendance arising from the current study was the pre-existing receipt of support from a primary care physician. This finding is congruent with the ‘*enabling factors’* category of ABM which outlines that the availability and nature of support can facilitate or impede health service utilisation [19]. However, it contrasts with previous findings in the field which have found *lack* of support and *poor* physician-patient relationships as barriers to attendance [32]. Our findings may be explained by a study by Gucciardi and colleagues [33] which found that some individuals perceive primary care physicians to provide the same coverage of diabetes education and lifestyle modification skills training as DSME. Similarly, another study found that individuals reported missing diabetes clinic appointments because they were already seeing a family physician or diabetes specialist [34]. These findings suggest that individuals who already feel well looked after and monitored may have their needs being met already and therefore, do not need to attend DSME. In such a situation, it may be beneficial for the individual, using their knowledge of their type 2 diabetes and self-management behaviours, and the physician, using their knowledge of DSME, to work together to consider whether DSME can offer any additional benefits over and above their pre-existing care and support – and whether such benefits are worth the costs of attending (e.g. time, finance).

The low prioritisation of one’s type 2 diabetes was also found to be a reason for non-attendance among some individuals. This is in line with the ‘*predisposing factors’* construct of ABM which outlines that attitudes towards one’s health and service use can facilitate or impede health service utilisation [19]. Attributing a low priority towards one’s type 2 diabetes has been cited as a key reason for non-attendance elsewhere in the literature [30, 35].

In the current study, low prioritisation of one’s type 2 diabetes was evident among individuals with a comorbidity whereby they perceived some conditions as more serious than others and prioritised another health condition over their type 2 diabetes. This supports similar findings that the burden associated with having and managing a comorbidity can impede resources and motivation to manage one’s type 2 diabetes [36], particularly when the disease and treatment burdens are greater for the comorbid condition than the burdens of type 2 diabetes [37, 38].

Disease and treatment burden from comorbid conditions can also have significant detrimental impacts on mental health and wellbeing [39]. This emphasises the need to acknowledge (and manage) such comorbidities when individuals are referred to DSME – particularly given associations between greater life satisfaction and wellbeing and improved treatment adherence [40].

The low prioritisation some people give their type 2 diabetes may also reflect a culture that perceives type 2 diabetes to be the ‘mild’ form of diabetes, despite its high morbidity and mortality rates [41]. The experiences of individuals in other cultures may differ depending on different cultural perceptions of type 2 diabetes. These findings suggest that health professionals may wish to raise awareness of the complications that could arise from their type 2 diabetes and help facilitate healthy behaviours that can benefit both their type 2 diabetes and comorbidities. Motivational interviewing which involves health professionals working alongside individuals to explore their views about their condition and behaviours, rather than immediately giving advice or referring them to DSME, may also help to better understand the unique views, needs and capabilities of the individual with comorbidity and assess whether DSME would be beneficial [42, 43]. Motivational interviewing can also be used to help people reflect on any ambivalence towards self-management and to help people prioritise what is most important to them in their lives [44].

Limited knowledge about the content, purpose and benefits of SDE also acted as a barrier to attendance. This finding is in line with the ‘*enabling factors’* construct of ABM which outlines that inadequate personal resources, such as limited knowledge about a service, can impede health service utilisation [19]. It also corroborates previous qualitative literature which cite a lack of familiarity and knowledge about DSME as common barriers to attendance [45]. A novel finding in this study was that individuals often attributed limited information about DSME to their primary care physician. Interestingly, however, several studies have found that physicians may feel unable to discuss the benefits, goals and expectations of DSME due to their own lack of knowledge of DSME [46, 47]. This highlights the need to empower individuals with type 2 diabetes to seek information about DSME themselves, rather than relying on their primary care physician as a main source of knowledge. Physicians who have a basic understanding of key sources where people can go to find out more information about DSME would likely be beneficial [47].

Previous research has also highlighted that a lack of familiarity and knowledge about DSME may stem from information about DSME that is inaccessible [47]. It is therefore crucial that information about DSME, as well as the programme itself, is made available in accessible and acceptable formats such as other languages, easy-read, British Sign Language and audio. The provision of DSME in other languages is highly relevant given that the prevalence of type 2 diabetes is three to five times higher in minority ethnic groups compared to the white British population and approximately 10–12 years earlier onset [48]. There is also strong evidence that health literacy – a person’s ability to understand and use information to make decisions about their health – is a strong predictor of health-related knowledge, illness self-management, health service use, health, and survival in people with type 2 diabetes [49]. People with low health literacy levels are more likely to have lower health-related knowledge [50, 51] and self-management [52, 53]. It is therefore critical that information about DSME (e.g. aims, benefits etc.) is provided in a format that is accessible and engaging to people with varying levels of health literacy [54]. The need to adopt and provide an adequate service for all societal groups also supports why a person-centred approach to overcoming barriers and increasing engagement with DSME is of paramount importance.

The final barrier to attending DSME identified in this study were the practical barriers associated with attending, namely conflicts between the programme’s schedule and other commitments and transportation issues with getting to the venue. Both of these barriers have been cited in previous literature [14] and are in line with the ‘*enabling factors’* category of ABM which outlines that practical barriers to obtaining care (e.g. income, other responsibilities) can impede health service utilisation [19].

Alternative methods of delivering DSME, such as offering the course online or via a mobile application, was well-received by participants and may help to alleviate barriers of time, commitment, mobility and transportation by enabling people to access DSME at their own convenience. This may be particularly pertinent for individuals who work shifts or on zero-hour contracts where it may be difficult or not possible for them to get paid time off work to attend DSME. Indeed, such individuals tend to be those with lower socio-economic status and, in turn, also at risk of poorer self-management outcomes [55]. An additional benefit of online DSME is that it can also be made available to large numbers of individuals at minimal cost [56]. An online DSME programme, called X-PERT Health, has demonstrated significant benefits to a range of diabetes outcomes, such as glycaemic control, body weight, blood pressure and cholesterol [57]. Similarly, in a randomised control trial, a web-based self-management programme for people with type 2 diabetes (called HeLP-Diabetes) improved glycaemic control over 12 months [58]. The offer of online DSME may therefore help to increase attendance and retain individuals by offering DSME at a time and place that suits people’s individual needs.

However, it is important to recognise that some individuals, particularly those with very low socioeconomic status, may be unable or find it difficult to engage with online DSME due to an absence of internet and/or technology (e.g. laptop, tablet, smart phone). To overcome this, additional funding and resources would be beneficial in order to assist individuals in accessing online DSME (e.g. provision of a tablet, free Wi-Fi), or increasing the frequency of DSME so that barriers of time and the programme’s schedule are minimised [59].

A strength of this research is its use of ABM as a well-supported model to illuminate the barriers to attending DSME. All three constructs of ABM were congruent with individuals’ reasons for not attending DSME. In particular, ABM specifically distinguishes the influence that health professionals’ judgement of health status can have on someone’s decision to engage with a particular health service – a key finding in this study. Our research therefore supports ABM in helping to map out the reasons for non-attendance to DSME.

A key limitation is that participants were recruited by phoning eligible people and inviting them to participate. Some individuals who declined the invite to participate held highly negative attitudes towards their type 2 diabetes. For example, some said their type 2 diabetes was shameful, burdensome and that they did not want to talk about it. It is therefore important to be aware that the views of individuals with such attitudes towards their diagnosis may not be fully captured in this study, and that these negative attitudes towards one’s diagnosis may have been a barrier to attendance in themselves.

In summary, the reasons for not attending DSME found in this study are wide-ranging, complex and individualised, providing further support to existing literature on the barriers to DSME attendance. The findings mirrored the constructs in ABM indicating that ABM is a well-supported model to help map out the barriers to DSME attendance. There is no one-size-fits-all solution to increasing attendance. Instead, a more person-centred approach to understanding people’s experience, needs and capabilities is essential to help identify and overcome barriers to attendance. This is in line with the evidence-base that person-centred approaches to health care and promotion are highly effective at overcoming barriers and increasing engagement [60].

## Supplementary Information

**Additional file 1.**

## Data Availability

The datasets used and/or analysed during the current study are available from the corresponding author on reasonable request.

## Abbreviations

DSME: Diabetes self-management education
ABM: Andersen’s Behavioural Model of Health Service Utilisation
