# Supplementary material for: A qualitative study exploring the barriers to attending structured education programmes among adults with type 2 diabetes
Source: BMC Health Serv Res. 2022 Apr 30;22:584. doi: 10.1186/s12913-022-07980-w (PMC9059690; doi:10.1186/s12913-022-07980-w)
Supplement: Supplementary file 1 — Additional file 1. [file 12913_2022_7980_MOESM1_ESM.docx]

**Appendix A – COnsolidated criteria for REporting Qualitative research studies (COREQ): 32 item checklist**

| **No** | **Item** | **Guide questions/description** | **Page** |  |
| --- | --- | --- | --- | --- |
| **Domain 1: Research team and reflexivity** |  |  |  |  |
| Personal Characteristics |  |  |  |  |
| 1. | Interviewer/facilitator | Which author/s conducted the interview or focus group? | Page 9 |  |
| 2. | Credentials | What were the researcher's credentials? *E.g. PhD, MD* | Page 9 |  |
| 3. | Occupation | What was their occupation at the time of the study? | Page 9 |  |
| 4. | Gender | Was the researcher male or female? | Page 9 |  |
| 5. | Experience and training | What experience or training did the researcher have? | Page 9 |  |
| Relationship with participants |  |  |  |  |
| 6. | Relationship established | Was a relationship established prior to study commencement? | Page 10 |  |
| 7. | Participant knowledge of the interviewer | What did the participants know about the researcher? e*.g. personal goals, reasons for doing the research* | Page 10 |  |
| 8. | Interviewer characteristics | What characteristics were reported about the interviewer/facilitator? e.g. *Bias, assumptions, reasons and interests in the research topic* | Page 10 |  |
| **Domain 2: study design** |  |  |  |  |
| Theoretical framework |  |  |  |  |
| 9. | Methodological orientation and Theory | What methodological orientation was stated to underpin the study? *e.g. grounded theory, discourse analysis, ethnography, phenomenology, content analysis* | Page 10 |  |
| Participant selection |  |  |  |  |
| 10. | Sampling | How were participants selected? *e.g. purposive, convenience, consecutive, snowball* | Page 7 |  |
| 11. | Method of approach | How were participants approached? e*.g. face-to-face, telephone, mail, email* | Page 7 |  |
| 12. | Sample size | How many participants were in the study? | Page 7 |  |
| 13. | Non-participation | How many people refused to participate or dropped out? Reasons? | Page 7 |  |
| Setting |  |  |  |  |
| 14. | Setting of data collection | Where was the data collected? e*.g. home, clinic, workplace* | Page 7 |  |
| 15. | Presence of non-participants | Was anyone else present besides the participants and researchers? | Page 10 |  |
| 16. | Description of sample | What are the important characteristics of the sample? *e.g. demographic data, date* | Page 8 and table 1 |  |
| Data collection |  |  |  |  |
| 17. | Interview guide | Were questions, prompts, guides provided by the authors? Was it pilot tested? | Page 10 & Appendix B |  |
| 18. | Repeat interviews | Were repeat interviews carried out? If yes, how many? | Page 10 |  |
| 19. | Audio/visual recording | Did the research use audio or visual recording to collect the data? | Page 10 |  |
| 20. | Field notes | Were field notes made during and/or after the interview or focus group? | Page 10 |  |
| 21. | Duration | What was the duration of the interviews or focus group? | Page 10 |  |
| 22. | Data saturation | Was data saturation discussed? | Page 11 |  |
| 23. | Transcripts returned | Were transcripts returned to participants for comment and/or correction? | Page 10 |  |
| **Domain 3: analysis and findings** |  |  |  |  |
| Data analysis |  |  |  |  |
| 24. | Number of data coders | How many data coders coded the data? | Page 11 |  |
| 25. | Description of the coding tree | Did authors provide a description of the coding tree? | Page 11 |  |
| 26. | Derivation of themes | Were themes identified in advance or derived from the data? | Page 10 |  |
| 27. | Software | What software, if applicable, was used to manage the data? | Page 10 |  |
| 28. | Participant checking | Did participants provide feedback on the findings? | Page 11 |  |
| Reporting |  |  |  |  |
| 29. | Quotations presented | Were participant quotations presented to illustrate the themes / findings? Was each quotation identified? e*.g. participant number* | Page 12-18 |  |
| 30. | Data and findings consistent | Was there consistency between the data presented and the findings? | Page 11-18 |  |
| 31. | Clarity of major themes | Were major themes clearly presented in the findings? | Page 11-18 and table 2 |  |
| 32. | Clarity of minor themes | Is there a description of diverse cases or discussion of minor themes? | Page 11-18 |  |

**Appendix B – Interview Schedule**

Section 1: Introduction

- First of all, could you please tell me a bit about your type 2 diabetes?
  - Prompts: causes, onset (when, prolonged vs sudden), initial symptoms, any difficulties, impact on life?
- Recently you were referred onto the X-PERT diabetes programme. Before having been referred onto this programme, had you engaged with any other formal programmes for your type 2 diabetes?
  - If yes…
    - When did you partake in this programme?
    - What programme was it?
    - Did you complete the full programme? (If no, then why?)
    - Were you referred by your GP or did you self-refer?

Section 2: Barriers to attending SDE

- Recently you were referred onto the X-PERT diabetes programme. May I ask who referred you onto the programme?
  - Prompts: self, GP, nurse
- What was your initial reaction to having been referred onto the X-PERT diabetes programme?
  - Prompts: feelings (happy/sad/pleased/shocked/motivated), any expectations?
  - Why do you think you felt this way?
- After having been referred/self-referred to X-PERT diabetes you later declined to take part in the programme. May I ask what made you decline?
  - Prompts: time constraints, stress, transportation problems, functional limitations, lack of interest, insufficiently informed, anxiety, worry
- How do you feel about not having attended?

Section 3: Programme improvements

- Under what circumstances would you have attended X-PERT diabetes?
  - What would have encouraged you to go at the time you turned it down?
  - Prompts: more time, better transportation, less worry/anxiety, more information, more interest, more motivation?
- Is there anything you think health professionals could have done differently to encourage you to attend the X-PERT diabetes course?
  - Prompts: promote benefits of attending, change location, facilitator, group vs individual setting, mode of delivery (face-to-face vs online), referral process?

Section 4: wrapping-up

- Is there anything else that you would like to share about not attending the X-PERT diabetes programme?
- Is there anything you feel is really important for us to know?

Thank you so much for your help. It’s been really interesting for me. To finish, could I just ask you some quick questions about yourself? They won’t be used in a way that could identify you.

- How old are you?
- How long have you been diagnosed with type 2 diabetes for?
- Do you know your height and weight?
- Do you know your BMI?

Thank you very much for participating. (Debrief and offer debrief form.)
